# Substrate–Enzyme Interactions in Intramembrane Proteolysis: γ-Secretase as the Prototype

**DOI:** 10.3389/fnmol.2020.00065

**Published:** 2020-05-19

**Authors:** Xinyue Liu, Jing Zhao, Yingkai Zhang, Iban Ubarretxena-Belandia, Scott Forth, Raquel L. Lieberman, Chunyu Wang

**Affiliations:** ^1^Center for Biotechnology and Interdisciplinary Studies, Rensselaer Polytechnic Institute, Troy, NY, United States; ^2^Department of Chemistry, New York University, New York, NY, United States; ^3^Instituto Biofisika (UPV/EHU, CSIC), University of the Basque Country, Leioa, Spain; ^4^Ikerbasque, Basque Foundation for Science, Bilbao, Spain; ^5^Department of Biological Sciences, Rensselaer Polytechnic Institute, Troy, NY, United States; ^6^School of Chemistry and Biochemistry, Georgia Institute of Technology, Atlanta, GA, United States; ^7^Department of Chemistry and Chemical Biology, Rensselaer Polytechnic Institute, Troy, NY, United States

**Keywords:** I-CLiPs, γ-secretase, substrate, interaction, Alzheimer’s disease

## Abstract

Intramembrane-cleaving proteases (I-CLiPs) catalyze the hydrolysis of peptide bonds within the transmembrane regions of membrane protein substrates, releasing bioactive fragments that play roles in many physiological and pathological processes. Based on their catalytic mechanism and nucleophile, I-CLiPs are classified into metallo, serine, aspartyl, and glutamyl proteases. Presenilin is the most prominent among I-CLiPs, as the catalytic subunit of γ-secretase (GS) complex responsible for cleaving the amyloid precursor protein (APP) and Notch, as well as many other membrane substrates. Recent cryo-electron microscopy (cryo-EM) structures of GS provide new details on how presenilin recognizes and cleaves APP and Notch. First, presenilin transmembrane helix (TM) 2 and 6 are dynamic. Second, upon binding to GS, the substrate TM helix is unwound from the C-terminus, resulting in an intermolecular β-sheet between the substrate and presenilin. The transition of the substrate C-terminus from α-helix to β-sheet is proposed to expose the scissile peptide bond in an extended conformation, leaving it susceptible to protease cleavage. Despite the astounding new insights in recent years, many crucial questions remain unanswered regarding the inner workings of γ-secretase, however. Key unanswered questions include how the enzyme recognizes and recruits substrates, how substrates are translocated from an initial docking site to the active site, how active site aspartates recruit and coordinate catalytic water, and the nature of the mechanisms of processive trimming of the substrate and product release. Answering these questions will have important implications for drug discovery aimed at selectively reducing the amyloid load in Alzheimer’s disease (AD) with minimal side effects.

## Four Classes of Intramembrane-Cleaving Proteases

Intramembrane-cleaving proteases (I-CLiPs, also called IMPAS) carry out regulated intramembrane proteolysis (RIP). They hydrolyze peptide bonds buried inside the membrane lipid bilayer (Brown et al., 2000) and release bioactive fragments (Haze et al., 1999; Niwa et al., 1999; Lal and Caplan, 2011; Lichtenthaler et al., 2011). Numerous I-CLiP substrates have been discovered, including the sterol regulatory element-binding proteins (SREBPs; Brown and Goldstein, 1997), the membrane receptor Notch (Selkoe and Kopan, 2003), and the amyloid precursor protein (APP; Annaert and De Strooper, 1999). I-CLiPs therefore play crucial roles in a variety of biological processes, including embryonic development, immune responses, and normal function of the nervous system. In addition, I-CLiPs contribute to many diseases such as cancer and Alzheimer’s disease (AD; Winter-Vann and Casey, 2005; Lichtenthaler et al., 2011; Düsterhöft et al., 2017).

Based on their catalytic mechanisms, I-CLiPs are classified into four families: rhomboid serine proteases (Wu et al., 2006), S2P-metalloproteases (Feng et al., 2007), di-aspartyl proteases (Fluhrer et al., 2009), and glutamyl proteases (Manolaridis et al., 2013). Although six classes of soluble proteases are known, I-CLiPs using cysteine or threonine as catalytic residue have not yet been identified. In the 3D structures of I-CLiPs, the polar catalytic residues are located well below the membrane surface, shielded from hydrophobic membrane environment by surrounding transmembrane helices (TMs), whereas water molecules are readily accesible to the catalytic residues through a hydrophilic chamber or channel.

### Serine I-CLiPs

**Figure 1:**
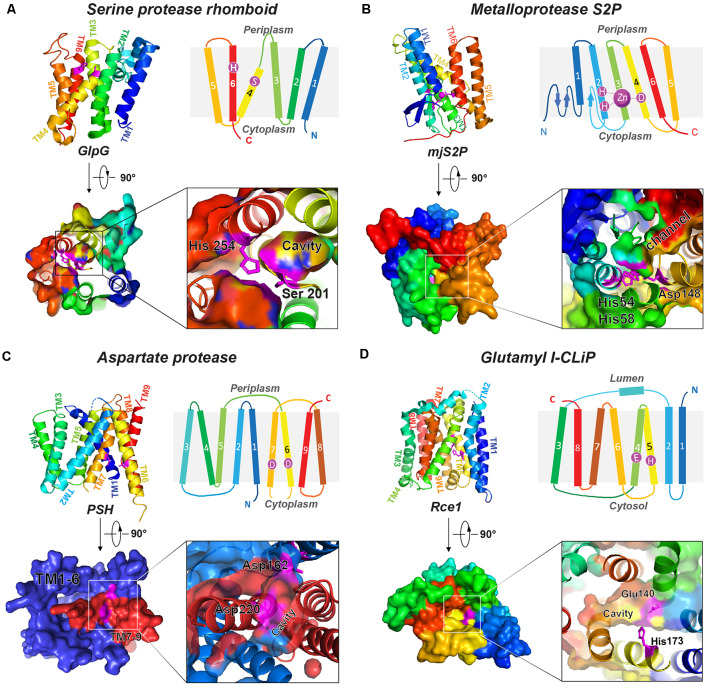
Representative structures of four I-CLiP families. Catalytic residues are labeled on the schematic structure, and the catalytic cavities are shown in the crystal structure from either the extracellular (or luminal) side or cytoplasmic side. **(A)** Serine protease rhomboid (GlpG, PDB: 2NRF). **(B)** Metalloprotease S2P (mjS2P PDB: 3B4R). **(C)** Aspartate protease MCMJR1, aka presenilin homolog (PSH, PDB: 4HYG). **(D)** Glutamyl I-CLiP (mmRce1 PDB: 4CAD).

Rhomboids constitute a large superfamily of serine I-CLiPs, which are involved in developmental signaling in *Drosophila* (Wasserman and Freeman, 1997), host invasion of protozoan parasites (Sibley, 2013), and human diseases such as cancer and neurodegeneration (Bergbold and Lemberg, 2013; Düsterhöft et al., 2017). Rhomboids have been intensely studied as model I-CLiP and also for their biological importance (see an excellent review by Strisovsky et al., 2009; Tichá et al., 2018). The rhomboid fold is composed of six TMs named TM1 to TM6 (Figure 1A). The catalytic dyad, serine (on TM4) and histidine (on TM6), is located at a V-shaped cavity accessible to the aqueous phase at a distance of 10–12 Å below the membrane surface (Wang et al., 2006; Wu et al., 2006; Ben-Shem et al., 2007; Figure 1A). During intramembrane proteolysis, the histidine activates the catalytic serine for a nucleophilic attack on substrates (Lemieux et al., 2007). Rhomboids recognize the helical TMs and a linear segment adjacent to the TMs of their substrates (Strisovsky et al., 2009). Structural and modeling studies proposed that the TMs of the substrates may bind the rhomboid at the interface of TM2 and TM5, where TM5 plays the role of the substrate gate (Baker et al., 2007; Xue and Ha, 2013; Zoll et al., 2014; Shokhen and Albeck, 2017). Binding studies reveal a role of allostery in catalysis. Dimerization of rhomboids is required for the formation of an exosite and subsequent allosteric substrate binding and activation (Arutyunova et al., 2014).

### Metalloproteases

**Figure 2:**
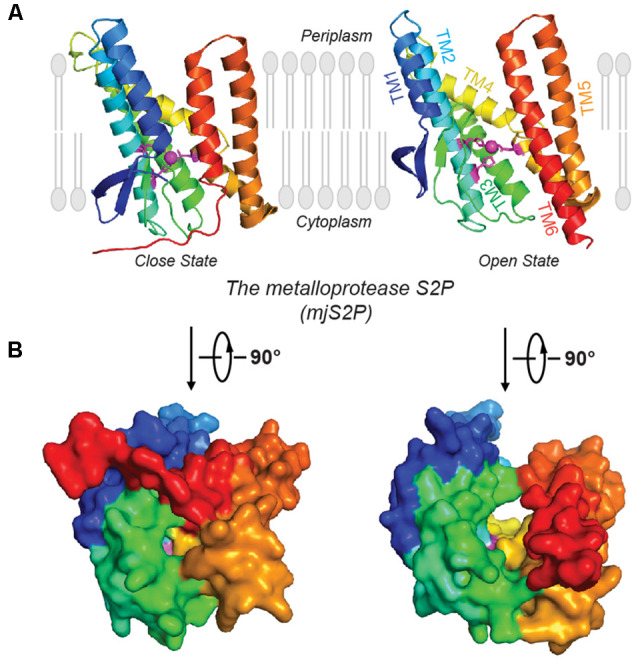
Open and closed conformations of mjS2P. **(A)** Crystal structures of the closed and open states of mjS2P, a metallo I-CLiP, and **(B)** cytoplasmic view of the catalytic cavity in the closed state and the cleft in the open state.

Site-2 proteases (S2Ps) constitute another family of metalloproteases, which activate membrane-bound transcription factors through RIP. S2Ps have been well studied in the context of cholesterol metabolism, with a zinc ion at its active site (Sun et al., 2016). After site-1 protease (S1P) cleavage, S2P cleaves SREBPs. The N-terminus of SREBP is then released and enters the nucleus to activate genes for biosynthesis and uptake of cholesterol (Sakai et al., 1996; Brown and Goldstein, 1997). An X-ray structure of *Methanocaldococcus jannaschii* S2P (mjS2P; Figure 1B), an S2P ortholog, revealed six TMs and three β-strands. The zinc ion, ~14 Å below the membrane surface, is coordinated by two histidine residues in an HEXXH motif (“H” is histidine, “E” is glutamate, and “X” is any amino acid) in TM2 and an aspartate in TM4 (Feng et al., 2007). Two conformations were identified: an open state and a closed state (Figure 2A). In the closed conformation, water accesses zinc *via* a polar channel open to the cytoplasmic side. In the open conformation, the TM1 and TM6 are separated by 10–12 Å, forming a cleft for substrate entry and positioning the catalytic zinc towards the substrate (Figure 2B).

### Di-Aspartyl Proteases

Di-aspartyl intramembrane proteases are characterized by a pair of catalytic aspartates. One of their catalytic aspartates is contained within the signature GXGD motif (“G” is glycine, “X” is any amino acid, and “D” is aspartate; Steiner et al., 2000; Fluhrer et al., 2009). Di-aspartyl intramembrane proteases are involved in many fundamental processes such as cell differentiation, development, immune surveillance, and virus maturation. This family has two key members: presenilin (PS) and signal peptide peptidase (SPP; Weihofen et al., 2002). PS is the catalytic subunit of γ-secretase (GS; Wolfe et al., 1999; Li et al., 2000), which cleaves Notch and APP transmembrane domain (TMD; Francis et al., 2002; Haass and Steiner, 2002), among over 90 substrates (Beel and Sanders, 2008). PS homologs (PSHs) can also cleave APP at the two major cleavage sites of PS (Torres-Arancivia et al., 2010; Naing et al., 2018a): the γ-site and the ε-site, generating Aβ42 and Aβ48, respectively (Naing et al., 2018a). A ~3.3-Å resolution crystal structure of an ortholog from *Methanoculleus marisnigri* (MCMJR1) showed nine TMs (Figure 1C) with TM1–TM6 equivalent to the amino-terminal fragment [N-terminal fragment (NTF)] and TM7–TM9 equivalent to the C-terminal fragment (CTF) of PS formed by autoproteolysis of GS (Li et al., 2013). TM1–TM6 tilt at angles of 15–35° away from the lipid membrane surface and form a horseshoe-shaped structure surrounding the CTF TMs. The active site aspartates (Asp 162 on TM6 and Asp 220 on TM7) are located in a cavity accessible from the cytoplasmic side, approximately 8 Å from the membrane surface. The structure of MCMJR1 characterized by small angle neutron scattering (SANS) is smaller than the crystal structure, indicating that the enzyme may be more compact in solution (Naing et al., 2018b).

### Glutamyl Proteases

**Figure 3:**
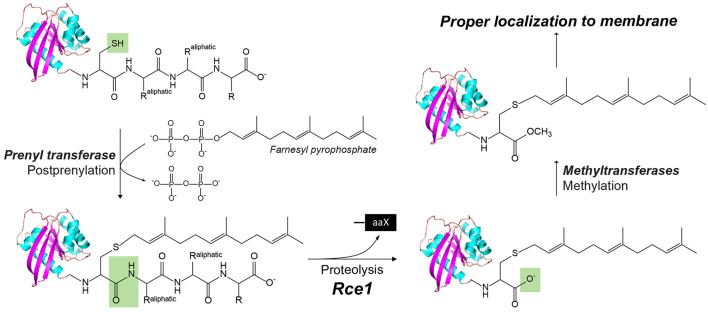
The posttranslational modification of proteins with a C-terminus CAAX motif by Rce1, a glutamyl IMP. In CaaX, “C” is cysteine, “A” is an aliphatic amino acid, and “X” is any amino acid. The posttranslational modifications of CAAX proteins include the cysteine isoprenylation, the −aaX release, and carboxyl methylation of the exposed isoprenylcysteine. The Rce1 is the prenyl endopeptidase for the release of the C-terminal −aaX peptide. These modifications are required for proper localization of the Ras to the membrane.

Ras converting enzyme 1 (Rce1) is a glutamate intramembrane protease (Manolaridis et al., 2013) found in the endoplasmic reticulum. Rce1 carries out posttranslational modifications of proteins with a C-terminus CAAX motif (“C” is cysteine, “A” is an aliphatic amino acid, and “X” is any amino acid residue; Figure 3; Boyartchuk et al., 1997). Substrates of Rce1 include Ras and prelamin A. Rce1 cleavage of these substrates is necessary for their function. The posttranslational modifications of CAAX proteins include cysteine isoprenylation, −AAX release, and methylation of the exposed C-terminal carboxyl of isoprenylcysteine (Figure 3; Schmidt et al., 1998). The Rce1 is the prenyl endopeptidase responsible for the release of the C-terminal −AAX peptide. These modifications are required for proper localization of the Ras protein (Michaelson et al., 2005) and can affect various signaling pathways during differentiation, proliferation, and oncogenesis (Winter-Vann and Casey, 2005; Christiansen et al., 2011). A crystal structure of the Rce1 ortholog from *Methanococcus maripaludis* (MmRce1) reveals eight TMs (Figure 1D; Manolaridis et al., 2013). TMs 4–7 form a conical cavity with an opening towards the cytosol, allowing solvent access and prenylated substrate accommodation. The catalytic dyad, a glutamate and a histidine, is located in the cavity approximately 10 Å away from the membrane surface.

Finally, a hybrid I-CLiP, ZMPSTE24, is a zinc metalloprotease that matures lamin A, a nuclear scaffold protein, through recognizing a CAAX motif (Pendás et al., 2002). Mutations in ZMPSTE24 are associated with premature aging, such as in Hutchinson–Guilford progeria syndrome (HGPS; Navarro et al., 2014). ZMPSTE24 resides in the inner nuclear membrane and is also known as farnesylated-protein converting enzyme 1 (FACE-1), and Ste24 in yeast. After farnesylation of the C-terminal CAAX motif, prelamin A is cleaved by either Rce1 or ZMPSTE24, and then the C-terminal cysteine residue is carboxymethylated (Figure 3). ZMPSTE24 further cleaves a 15-residue CTF, resulting in mature lamin and its release from the nuclear membrane. In progeroid conditions caused by ZMPSTE24 mutation, farnesylated and methylated prelamin accumulates in the nuclear membrane. ZMPSTE24 contains an extraordinary intramembrane chamber, large enough to accommodate a ~10-kDa protein or ~450 water molecules (Pryor et al., 2013). The active site residues are facing the chamber, with an arrangement almost identical to bacterial thermolysin.

## Gs in Health and Disease

GS is reported to cleave over 90 substrates (Beel and Sanders, 2008). Conversely, aberrant GS cleavage is associated with many diseases, including cancer, skin disorder, and neurodegenerative diseases (Shih and Wang, 2007; Kelleher and Shen, 2010). Here, we highlight the two most prominent GS substrates, APP and Notch, which are involved in AD and cancer, respectively.

### GS and AD

Amyloid plaques are a hallmark of AD pathology, which are mainly composed of aggregated amyloid-β (Aβ) peptides. Aβ deposits have been proposed as the initial trigger in the decade-long progression towards neurodegeneration in AD (Tanzi and Bertram, 2005), which leads to tau pathology and eventually widespread neuroinflammation. Aβ peptides are produced from APP by the consecutive action of two proteases, β-secretase and GS. β-Secretase sheds the ectodomain of APP, generating C99 and the N-terminus of the subsequent Aβ species (Mullard, 2017). GS is the I-CLiP that cleaves within the TM of APP (APPTM), releasing different lengths of Aβ peptides into the extracellular matrix or endosome lumen (Qi-Takahara et al., 2005; Takami et al., 2009). Longer Aβ peptides (e.g., Aβ42 and Aβ43) are particularly prone to aggregation.

**Figure 4:**
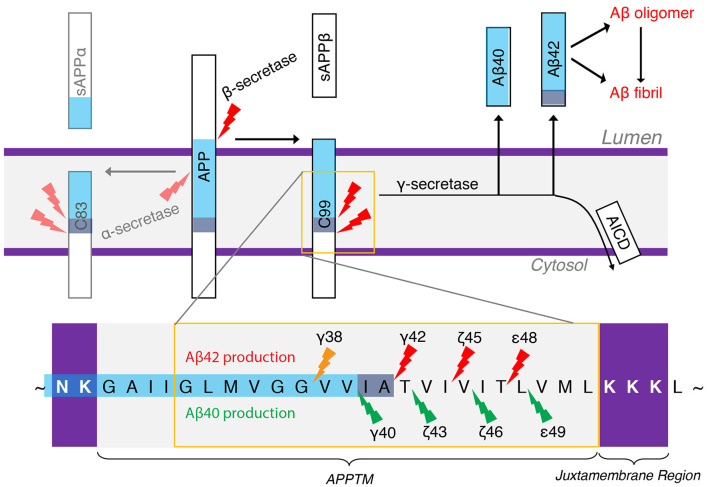
The generation of Aβ40 and Aβ42 from amyloid precursor protein (APP). α-Secretase and β-secretase are the sheddases generating C83 and C99 from APP, respectively. γ-secretase (GS) is the I-CLiP that carries out intramembrane proteolysis of C99 to generate Aβ, a pathogenic peptide in Alzheimer’s disease (AD).

There are two APP processing pathways (Figure 4). In the non-amyloidogenic pathway, APP is first cleaved by α-secretase to generate C83, and further cleavage of GS can no longer generate Aβ. In the amyloidogenic pathway, APP is first cleaved by β-secretase to generate a membrane-bound CTF containing 99 amino acid residues (C99). C99 is then the substrate of GS to generate Aβ, the pathogenic peptide for AD (Lichtenthaler et al., 2011), while the APP intracellular domain (AICD) is liberated into the cytoplasm (Haass and Steiner, 2002). β-Secretase and GS both localize to the lipid rafts of cell or organellar membranes, and cholesterol plays an important role in the enzyme activity (Tun et al., 2002; Urano et al., 2005). The observation of different lengths of Aβ peptides suggests a successive C-terminal trimming mechanism of GS after the initial ε-cleavage (Qi-Takahara et al., 2005; Takami et al., 2009). In addition to Aβ40 and Aβ42, Aβ38, Aβ43, Aβ45, Aβ46, and Aβ48 are also identified. Starting from two initial ε-cleavage sites ε48 and ε49, Aβ40, Aβ43, and Aβ46 are generated from Aβ49 through successive shedding of tripeptides. Non-transitional state GS inhibitors (GSI), DAPT and Compound E, suppress intracellular Aβ40 production while increasing Aβ43 and in turn Aβ46 levels (Qi-Takahara et al., 2005). Aβ45, Aβ42, and Aβ38 are generated from Aβ48 (Figure 4). These two product lines have been established using LC–MS/MS (Takami et al., 2009). The stepwise cleavage sites are named ε48/ε49, ζ45/ζ46, ζ42/ζ43, and γ38/γ40 (Lichtenthaler et al., 2011; De Strooper and Chávez Gutiérrez, 2015; Langosch and Steiner, 2017).

Aβ peptides can aggregate into oligomers and fibrils. Longer Aβ forms, such as Aβ42 and Aβ43, are especially prone to aggregation and are therefore much more toxic (Makin, 2018). Mutations in APP on chromosome 21q (Levy et al., 1990; Goate et al., 1991; Tanzi and Bertram, 2005; Bertram et al., 2010) and in PS 1 and 2 genes (PSEN1 and PSEN2, respectively) on chromosomes 14 and 1 (Levy-Lahad et al., 1995; Rogaev et al., 1995; Sherrington et al., 1995) can cause early-onset familial Alzheimer’s disease (FAD), characterized by an increased Aβ42/Aβ40 ratio biochemically. The most common FAD mutations occur in PS, underlining the important biological role for GS. The successive cleavage of the APP substrates progressively destabilizes the GS–Aβ_n_ complex with the shortening of the Aβ_n_. It has been shown that PSEN mutations will further destabilize the Aβ_n_–GS complex, resulting in the release of longer Aβ_n_ (Szaruga et al., 2017) and raising the Aβ42/Aβ40 ratio.

**Figure 5:**
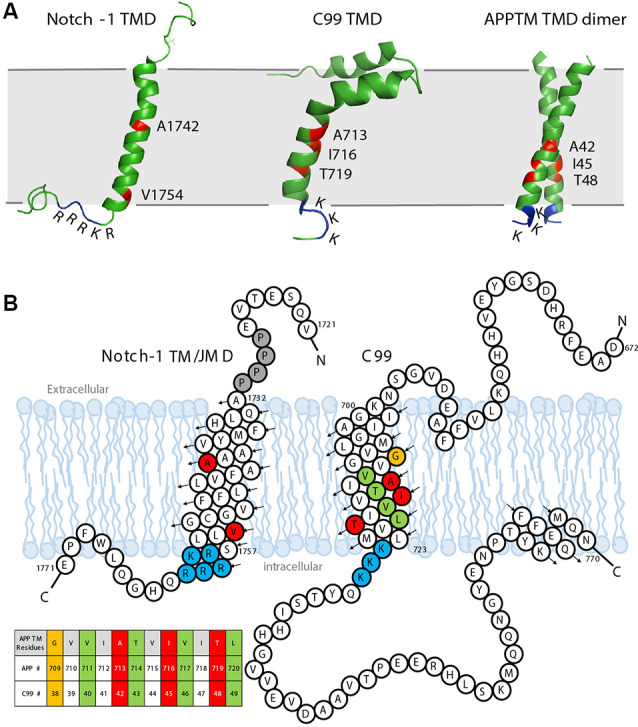
The sequence and solution structure of the transmembrane domain (TMD) of Notch-1 and APP. **(A)** The solution nuclear magnetic resonance (NMR) structure of Notch-1 TMD (PDB: 5KZO), C99 TMD (PDB: 2LP1), and APPTM TMD dimer (PDB: 2LZ3). The major cleavage S3 site on Notch-1 and Aβ42 cleavage sites on C99/APPTM are labeled red. The juxtamembrane domains lysine (K) and arginine (R) are indicated. **(B)** The sequence and topology of Notch-1 TMD (adapted with permission from Deatherage et al., 2017; copyright 2017 American Association for the Advancement of Science) and APP-C99 (adapted with permission from Beel et al., 2008; copyright 2008 American Chemical Society). Black arrows indicate the direction of the helices from N- to C-termini. Important residues are color coded: the positively charged juxtamembrane domain residues are labeled blue. In Notch-1, the major S3 cleavage site Val1754 and major S4 cleavage site A1742 are labeled red. In APP-C99, the Aβ40 cleavage sites are labeled green, and the Aβ42 cleavages sites are labeled red/orange. A table of APPTM residue numbering is provided in the context of both APP and C99.

As a GS substrate, the local conformation and dynamics of APPTM contribute to the observed cleavage sites. A right-handed APPTM helical dimer was characterized by nuclear magnetic resonance (NMR) in solution (Figure 5A). In the same study, FAD mutations V44M and V44A within APPTM were found to selectively expose the T48 site for fast solvent exchange. This may promote T48 for the initial ε-cleavage over L49 and consequently shift cleavage preference towards Aβ42 production (Chen et al., 2014).

### GS and Notch Signaling

Notch signaling is involved in neurogenesis, synapse growth and plasticity, and neuronal death in vertebrates (Kopan and Ilagan, 2009). The Notch receptor is a single-span membrane protein like APP. For Notch-1, the TMD is from residues Ala1732 to Ser1757, terminated by a cluster of basic residues: _1758_RKRRR_1762_, similar to the APP intracellular juxtamembrane region _724_KKK_726_ (Deatherage et al., 2017; Figures 5A,B). In the Notch signaling pathway, Notch precursors are cleaved by a furin-like convertase at Site-1 (S1), generating the mature Notch receptor, a 2,500-residue membrane protein. The shedding of the Notch ectodomain following S1 cleavage is carried out by ADAM, a metalloprotease, which is referred to as Site-2 (S2) cleavage. After shedding, the Notch receptor undergoes cleavage by GS, which, like APP, is Processive (van Tetering and Vooijs, 2011). For Notch-1, the initial cleavage, which is called the Site-3 (S3) cleavage, mainly occurs at Val1754 (Figure 5B), releasing a large Notch intracellular domain (NICD; Deatherage et al., 2017). The NICD translocates to the nucleus, forming an activator complex (Kitagawa, 2015). The processive cleavage stops at Site-4 (S4), mainly at Ala1742, and an extracellular domain (ECD) peptide (Nβ) terminating at residue 1741 is released (Deatherage et al., 2017). PS1 mutations associated with FAD also cause a shift in the Nβ cleavage site, in a similar manner to Aβ (Okochi et al., 2006).

### Targeting GS for AD Drug Discovery

A major theme in AD drug discovery is to reduce amyloid by inhibiting GS. To date, however, clinical trials of GSIs have failed due to severe side effects and worsening cognitive functions in patients. The so-called Notch-sparing APP-selective inhibitors, which preferentially inhibit APP cleavage over Notch by GS, did not show reduced toxicity (Crump et al., 2012; Tong et al., 2012). Another strategy in AD drug discovery is to develop GS modulators (GSM), which bias GS activity towards generating shorter, less toxic Aβ peptides (Bursavich et al., 2016). Given the complexity of the role of GS in biology beyond Notch and APP, it is imperative that the molecular details of GS interactions with substrates be understood to inform an effective strategy for discovering disease-modifying drugs in AD.

## Structures of APO GS and its Subunits

**Figure 6:**
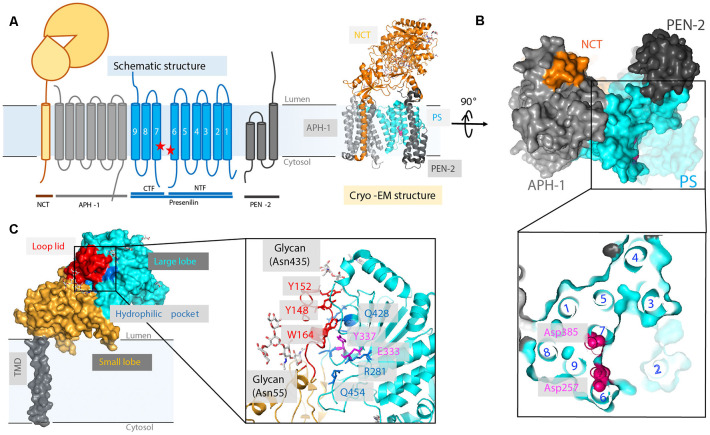
High-resolution cryo-electron microscopy (cryo-EM) structure of apo γ-secretase (GS; PDB: 5A63). **(A)** Schematics of GS complex. **(B)** The horseshoe shape arrangement of the GS TMs, with two catalytic aspartates located on the convex side of the transmembrane helix (TM) horseshoe: Asp257 on TM6 and Asp385 on TM7. The location of TM2 is drawn based on a bound-state GS structure (PDB: 5FN3). **(C)** The cryo-EM structure of a nicastrin subunit; a close-up view of the hydrophilic pocket is shown.

There are four essential components of GS: PS (also abbreviated as PSEN), nicastrin (NCT), anterior pharynx-defective 1 (APH-1), and PS enhancer 2 (PEN-2; Kimberly et al., 2003; Figure 6A). The catalytic subunit, PS, consists of nine TMs with two catalytic aspartates, Asp257 and Asp385, located in TM6 and TM7, respectively (Wolfe et al., 1999; Li et al., 2013; Bai et al., 2015b). GS is matured and activated only after PS undergoes autoproteolysis, cleaving itself between TM6 and TM7 and dividing PS into an NTF and a CTF (Thinakaran et al., 1996; Knappenberger et al., 2004). NCT, which has a large, heavily glycosylated ECD and a single TM segment (Xie et al., 2014), is involved in the initial binding of substrate and likely inhibits the docking of substrates with long N-termini prior to the action of a sheddase. APH-1 contains seven TMs and is mainly responsible for the assembly, scaffolding, and stabilization of the GS complex (Brunkan et al., 2005). PEN-2, composed of three TMs is required for PS autoproteolysis and stabilizes PS NTF and CTF (Luo et al., 2003; Prokop et al., 2004). Although these four components are sufficient for performing cleavage, additional proteins are possibly involved in the modulation of the GS cleavage activity (Wakabayashi et al., 2009). For example, TMP21, a member of the p24 cargo protein family, is reported to be a component of PS complexes and regulates GS cleavage (Chen et al., 2006).

### X-Ray Structure of a PSH From *Methanoculleus marisnigri* JR1 (MCMJR1)

A PS ortholog was discovered from *Methanoculleus marisnigri* JR1 (Torres-Arancivia et al., 2010), and its X-ray structure (Figure 1) was solved soon thereafter (Li et al., 2013). The two catalytic aspartate residues are ~9–10 Å apart. This distance is too far for the coordination of a catalytic water when compared to soluble aspartate proteases. In pepsin, the two catalytic aspartates are ~3 Å away from each other, and in HIV protease, the two catalytic aspartates are only 2.3 Å apart (Kovalevsky et al., 2007; Weber et al., 2013). Several explanations may account for the MCMJR1 structure being in an inactive conformation. Limited proteolysis was used during crystallization, which likely removed linker regions between TMs that in turn allow new motions to occur. Another possibility is that the apo state of the enzyme is an inactive conformation, and substrate binding triggers a conformational change to move the two aspartate residues closer together to carry out catalysis, as suggested by structures of GS (see below and Bai et al., 2015a).

### X-Ray Structure of the NCT Homolog From *Dictyostelium purpureum* (DpNCT)

The structure of NCT was first solved for a eukaryotic homolog from *Dictyostelium purpureum* (DpNCT), which shares 40% sequence identity with human NCT (HsNCT). The 1.95-Å resolution crystal structure reveals a large ECD and a single TM helix (Xie et al., 2014). The ECD of DpNCT contains a large lobe and a small lobe, interacting with each other through numerous van der Waals contacts at the center of the interface and 11 hydrogen bonds at the periphery of the interface. A pocket in the large lobe is surrounded by hydrophilic side chains, which may be responsible for anchoring hydrophilic N-termini of the substrates such as APP and Notch. An extended loop from the small lobe forms a lid that hovers above the pocket, likely gating substrate entry. Conformational changes are needed for substrate recruitment (Li et al., 2014; Xie et al., 2014).

### Cryo-Electron Microscopy Structure of GS

After intensive cryo-electron microscopy (cryo-EM) efforts (Lu et al., 2014; Sun et al., 2015), a 3.4-Å map of GS was obtained with excellent main-chain connectivity and discernable side-chain features (Bai et al., 2015b; Figures 6A,B). Among the 20 TMs identified, TM2 of PS1 shows the highest degree of flexibility. Except for TM2 and TM6, the other 18 TMs were observed with good side-chain density, including the seven TMs of APH-1, the other seven TMs of PS1, the three TMs of PEN-2, and the lone TM of NCT. Overall, the TMs form a horseshoe shape (Bai et al., 2015b; Sun et al., 2015), with PS1 and APH-1 at the center and PEN-2 and NCT at the tips of the horseshoe. The two catalytic residues (Asp257 and Asp385 of PS1) are on the convex side of the TM horseshoe (Figure 6B). The cryo-EM structure of PS solved here is largely superimposable with the PSH from MCMJR1. The ECD of NCT directly interacts with PEN-2. The TMs predominantly interact through van der Waals contacts among hydrophobic side chains.

**Figure 7:**
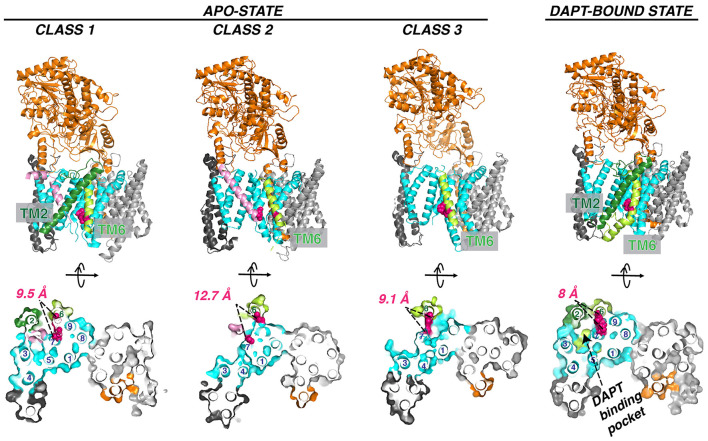
Three classes of apo GS conformation and DAPT-bound GS structure. The pink helix in class 1 (PDB: 5FN3) and class 2 (PDB: 5FN4) represents an unidentified substrate co-purified with GS. TM2 observed in class 1 and DAPT bound state (PDB: 5FN2) is in forest green. The two catalytic aspartate residues are colored red. Helix numbering of the PS1 subunit is labeled in the orthogonal view from the cytosolic side.

The flexibility of PS1 TM2 and TM6 seen in the cryo-EM structure suggests a pathway for the substrate entrance and conformational changes during substrate docking and translocation. Masked classification of the apo-state GS cryo-EM dataset revealed three major classes of conformations (Figure 7; Bai et al., 2015a). In class 1, TM2 from PS1 is ordered, and there is unassigned density corresponding to a kinked α-helix, which may be a fortuitously co-purified cellular substrate or product. In class 2, the TM2 helix could be also observed but not well defined. In class 3, no substrate or TM2 could be observed. PEN-2 rotates away from PS1, together with PS1 TM3 and TM4, while PS1 TM5/TM6 move towards the extracellular/lumenal space and TM6 rotates towards TM7. In the cryo-EM structure of GS in complexes with the peptidomimetic inhibitor DAPT (Bai et al., 2015a), the conformation of PS1 is very similar to class 1. Both PS1 TM2 and the linkers between TM2 and TM1 and TM2 and TM3 become ordered in the presence of DAPT, as well as part of the long linker between TM6 and TM7. TM6 displays a kink near the active site, forming a hydrophobic binding pocket with TM2, TM3, TM5, and TM7 for DAPT, the same pocket that APP and Notch substrates occupy revealed by later cryo-EM structures (see “Interaction of GS With Substrates” section). Crucial structural features and interactions of PS1 are listed in Table 1.

**Table 1:** Function and motifs in the transmembrane domains (TM) and loops (L) of PS1.

		Residue#	Motifs	Function	References
NTF	TM1	G78-V103	-	Interaction with NCT	Bai et al. (2015a,b)
	L1	S104-E123	-	Hydrophilic for substrate recognition	Takagi-Niidome et al. (2015)
	TM2	T124-C158	-	Most dynamic TM; lateral gating of TMD substrate entry	Bai et al. (2015a,b)
	TM3	Y159-A192	-	Interaction with PEN-2	Bai et al. (2015a)
	TM4	V193-G217	NF motif (_204_NF_205_)	Interaction with PEN-2	Kim and Sisodia (2005)
	TM5	P218-P242	-	Hot spot for FAD mutation	Bai et al. (2015a)
	TM6	E243-Q276	-	Lateral gating of TMD substrate entry	Bai et al. (2015a)
			Catalytic aspartate (D257)	Active site aspartate	Bai et al. (2015a,b)
	L6	E277-L381	Endoproteolysis region	γ-Secretase autocleavage site	Bai et al. (2015a)
CTF	TM7	G382-A398	GxGD motif (_382_GLGD_385_)	Peptide bond cleavage and substrate selectivity	Steiner et al. (2000)
	TM8	T399-K429	-	Interaction with APH-1	Bai et al. (2015a)
	TM9	K430-I467	PAL motif (_433_PAL_435_)	PS1 endoproteolysis and γ-secretase activity	Sato et al. (2008)
			Hydrophobic C-terminus (_465_FYI_467_)	Interaction with a hydrophobic pocket in APH-1	Bai et al. (2015a)

*Values for age represent the mean ± standard deviation. Odds ratios (O.R.) are normalized to *APOE*-ɛ3 and non-*APOJ*-C, making these values “1”. Risk scores shown are sums of the natural log of the odds ratios. Non-*APOE*-ɛ4 group includes *APOE*-ɛ2 carriers that have O.R. of 0.6*.

The cryo-EM structure of GS also reveals new details regarding HsNCT (Bai et al., 2015b; Figure 6C). First, the residues involved in GS substrate recognition, Glu333 and Tyr337, are located in a hydrophilic pocket. Charged arginine residues (Arg281, Arg285, Arg429, and Arg432) in this buried pocket may also mediate specific hydrogen bonding and salt bridges for substrate recruitment. Second, 11 glycosylation sites were identified on the large lobe. This heavy glycosylation likely contributes to substrate recruitment (Shah et al., 2005) and in ECD folding and stability. Two glycans on Asn55 and Asn435 from the large lobe flank the lid from the small lobe.

## Interaction of GS With Substrates

Several interaction models have been put forth to explain the successive cleavage of APP substrate by GS [see “GS and AD” section]. First, a “piston model” was proposed in which APP–C99 remains in a helical conformation but shifts successively downward towards the active site of PS (Takagi et al., 2010). However, downward shifting of the substrate may make it harder for the product to be released as processive cleavage progresses. Second, a substrate “bending model” was put forward based on C99 TM backbone dynamics and the bend of a co-purified substrate observed in the class I cryo-EM structure of GS. In this model, C99 presents the scissile bond by bending the TM helix (Scharnagl et al., 2014; Langosch et al., 2015). Lastly, as elaborated in this section, growing evidence supports a substrate TM unwinding model to generate the scissile peptide bond in extended conformation, favoring the extended β-strand conformation that binds productively to the active site of proteases (Madala et al., 2010).

### Docking Site Mapping by Nuclear Magnetic Resonance (NMR)

**Figure 8:**
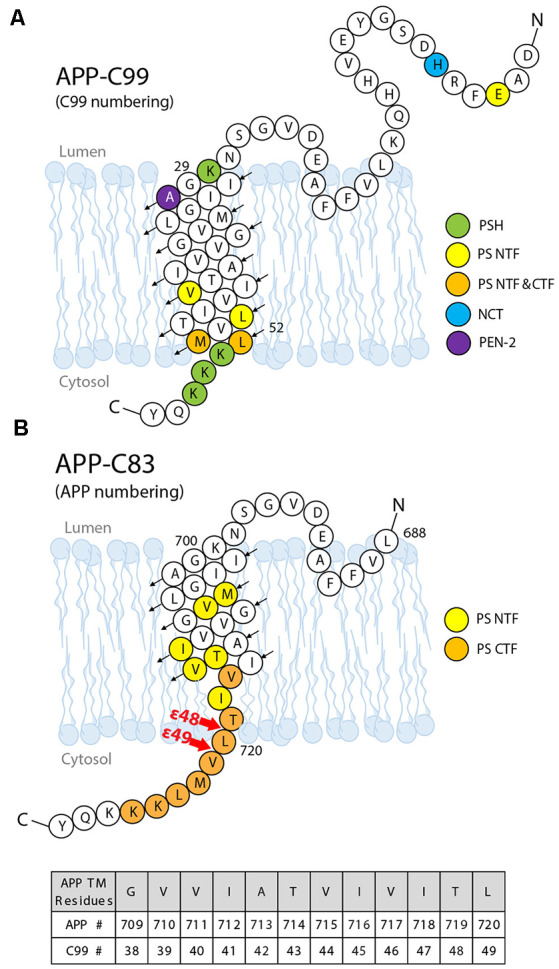
The substrate–enzyme interaction sites on the APP substrate. **(A)** Interaction sites identified by NMR titration (green) and photoaffinity cross-linking (other colors). **(B)** Interaction sites between the presenilin subunit and APP identified by cryo-EM. Upon I-CLiP binding, C-terminus unwinding occurs, and a β-strand is formed from L720 to K725.

Solution NMR has been utilized to probe substrate docking of APPTM, using PS orthologs that are catalytically active towards the TM segment of APP (APPTM). Chemical shift perturbation (CSP) showed that juxtamembrane regions of APPTM mediate its docking to MCMJR1. The largest CSP occurred at residues K28 and K54 of APPTM (Figure 8), likely mediating electrostatic interactions with the MCMJR1 (Clemente et al., 2018). Binding of the substrate to MCMJR1 decreased the magnitude of amide proton chemical shifts δ_H_ at the C-terminal half of the substrate APPTM. Because amide δ_H_ has a strong positive correlation with hydrogen bond strength, the pattern of decreasing δ_H_ indicates that the docking to the enzyme weakens helical hydrogen bonds and unwinds the substrate TM helix around the initial ε-cleavage site. The APPTM V44M substitution linked to FAD caused more CSP and helical unwinding around the ε-cleavage site. MAMRE50, another archaeal ortholog of PSH, which cleaved APPTM at a higher rate, also caused more CSP and helical unwinding in APPTM than in MCMJR1. These data suggest that docking of the substrate TM helix and helix unwinding are coupled in intramembrane proteolysis by PS and its ortholog, and FAD mutations can modify enzyme–substrate interaction.

### Interaction Mapping by Photoaffinity Cross-Linking

A comprehensive mapping of the interaction between APP C99 and GS at residue resolution was accomplished by photoaffinity mapping (Fukumori and Steiner, 2016). Sixty-eight His-tagged C99 constructs containing photo-active amino acid *para-*benzoyl-L-phenylalanine (Bpa) substitution, from residues D1 to D68, were produced. After incubation with CHAPSO-solubilized GS and UV irradiation, the Bpa residue photo-cross-linked with nearby GS residues, within ~3 Å. Cross-linked substrates and GS components were isolated by Ni-NTA affinity pulldown followed by dissociation of GS for photoaffinity mapping.

Photoaffinity mapping showed that APP C99 residues Val44, Leu49, Met51, and Leu52 are cross-linked to PS1 NTF, representing major substrate–enzyme interaction sites. Cross-linking at an exosite was also observed. C99 Glu3 was cross-linked to PS1 NTF, most likely through interaction with the loop L1 between TM1 and TM2. His6 and Ala30 cross-linked with NCT and PEN-2, respectively. Ala30 is not close to PEN-2 in the cryo-EM structure of the GS–APP complex, indicating that major conformation changes occur during substrate–GS interaction. Met51 and Leu52 also cross-linked to PS1 CTF (Figure 8), as expected. To distinguish between interactions for substrate recruitment and for cleavage, “substrate-binding chase” experiments were carried out: first, C99 “binding” and cross-linking to GS were performed at 4°C to inhibit enzyme cleavage, followed by a 37°C cleavage “chase” experiment. When the substrate was cross-linked with PS1 NTF, it could be cleaved under 37°C and could also be inhibited by GSIs. However, when the substrate and NCT/PEN-2 are cross-linked, the substrate cannot be cleaved, indicating that exosite cross-linking blocked the substrate passage from the GS exosite to the active site. Furthermore, the cross-linking of PS1 NTF was suppressed by GSIs while cross-linking involving PEN-2 was increased with GSI’s presence. These data further confirmed the existence of a substrate docking site distinct from the active site. In summary, these studies show that the GS substrate binds to GS in two steps: first, the substrate binds to the exosite, likely formed by NCT, PEN-2, and NTF, and then the substrate translocates to the active site formed by PS1 NTF/CTF. Compared with the interaction sites identified in the cryo-EM structure of the GS–APP complex (Zhou et al., 2019; Figure 8B), this photoaffinity mapping showed additional interaction sites during substrate docking and translocation.

### Biophysical Studies of Substrate TM Unwinding

Solid-state NMR revealed that the TM helix of C99 unravels downstream of the ε-sites (Sato et al., 2009). Under isotopic labeling, deep-ultraviolet resonance Raman (dUVRR) spectra of Gurken, a substrate for GlpG rhomboid and MCMJR1 (Torres-Arancivia et al., 2010), displays both α-helical and 3_10_-helical geometry; 3_10_-helical unwinding was observed during binding to the enzyme (Brown et al., 2018). When the 3_10_-helical content was suppressed using a proline-to-alanine mutation, binding was not affected, but cleavage was inhibited. This result is consistent with the fact that the initial docking site is distinct from the active site proposed for GS (Fukumori and Steiner, 2016) and rhomboids (Arutyunova et al., 2014). As mentioned above, hydrogen bond weakening and helical unwinding in the APPTM C-terminus upon binding to MCMJR1 were also observed in solution NMR (Clemente et al., 2018).

### Cryo-EM Structure of GS in Complex With Notch and APP

**Figure 9:**
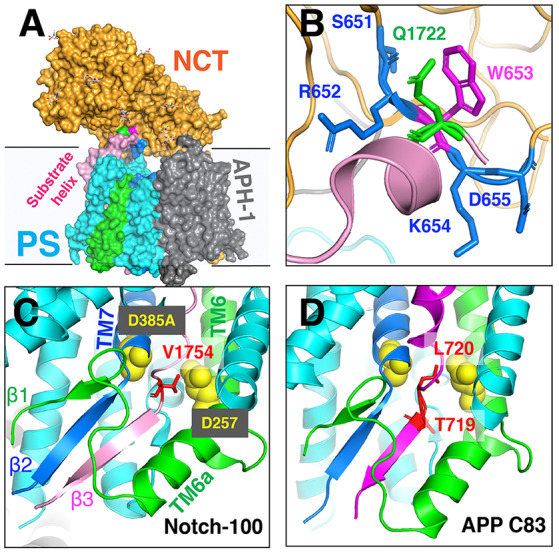
The cryo-EM structure of the GS–substrate complex with Notch-100 and APP-C83. Nicastrin (NCT) is colored orange and PS1 cyan. TM6 and TM7 from PS1 are colored green and blue, respectively. Substrates are in pink/magenta. **(A)** The overall complex structure. **(B)** A close-up view of the NCT hydrophilic pocket interacting with the Notch substrate. Q1722 is on Notch-100. 651SRWKD655 is on NCT. **(C)** The intermolecular β-sheet around Notch-100 C-terminal cleavage sites. TM6 extends to two helices (TM6/TM6a). The hybrid β-sheet consists of β1 from TM6, β2 from TM7, and β3 from the substrate. Two catalytic aspartates are at the S3 cleavage site. **(D)** A similar hybrid β-sheet between APP-C83 and PS TM6/TM7. The ε-cleavage sites are in extended conformation.

The unwinding of the substrate TM helix at the carboxyl terminus was confirmed in cryo-EM structures of human GS in complex with mouse Notch-100 (Yang et al., 2019) and APP-C83 fragment (Zhou et al., 2019). To stabilize the GS–substrate complexes, disulfide-cross-linked GS–APP/Notch complexes were generated with human GS containing an active site mutation (PS1-Q112C/D385A, PEN-2, APH-1aL, and NCT) and APP-C83 (V695C; Zhou et al., 2019) or Notch-100 (P1728C; Yang et al., 2019). In the highest-resolution (2.6–2.7 Å) complex structure, TM6 extends to having two helices (TM6 and TM6a; Figure 9); TM2 and the loop between TM6/TM7 of PS are more ordered compared to free GS (Bai et al., 2015b).

The structures reveal that the C-termini of both APP and Notch adopt a β-strand conformation, forming an intermolecular, antiparallel β-sheet with two induced β-strands from PS1 NTF (TM6) and CTF (TM7). In this β-strand mode, the cleavage sites on substrate TM are in a more extended conformation and become more exposed. The ε-cleavage sites (residues T719 and L720) in APPTM are fully extended (Figure 9D), as is the S3 cleavage sites (V1754) at the C-terminal part of Notch TM (Figure 9C).

Additional details of the participation of the NCT ECD in substrate recruitment (Xie et al., 2014) were revealed in the complex structures. In addition to the hydrophilic pocket reported in DpNCT (Xie et al., 2014), another hydrophilic pocket (Ser651, Arg652, Lys654, and Asp655) located at the small lobe near the membrane was identified (Figure 9A). A short helix of Notch-100 is inserted into the hydrophilic pocket (Yang et al., 2019). Kinetic data showed that the binding affinity between GS and Notch is driven by TMD interaction and that the affinity decreases with increasing ectodomain length and structure (Bolduc et al., 2016). Substrates with longer ectodomains could only be efficiently cleaved after disrupting the NCT fold. The sterical hindrance of NCT likely contributes to the selectivity of the GS substrate.

## Open Questions and Future Directions

**Figure 10:**
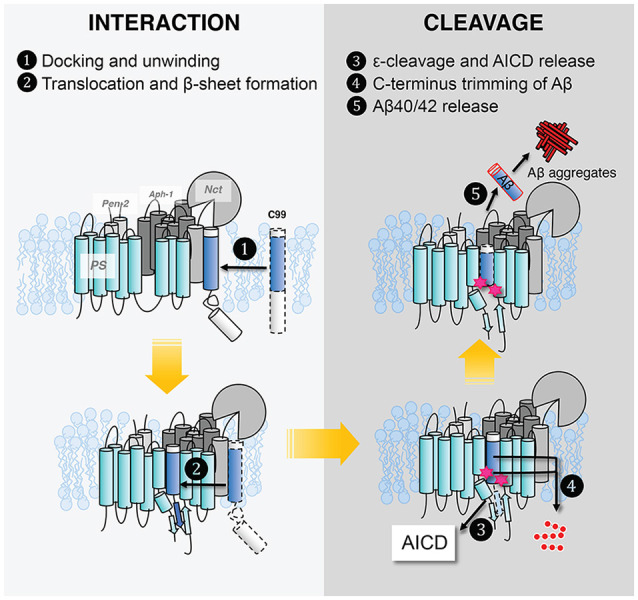
Five steps in γ-secretase–substrate interaction and cleavage to produce Aβ.

Despite the tremendous progress detailed above, our molecular picture of GS remains far from complete. We do not know the effect of the lipid composition of the lipid bilayer, hence how the cellular location of APP affects GS cleavage and how FAD mutations affect Aβ production and increase the Aβ42/Aβ40 ratio. We still do not have full clarity on how GS interacts with its substrate. In Figure 10, we outline the major steps of APP C99 interaction with GS, which ultimately results in the production of Aβ, a pathogenic peptide in AD. In each step, there are many important, unanswered questions:

1. C99 docks to the GS exosite, coupled with helical unwinding near the initial cleavage site (Clemente et al., 2018). However, we do not know the molecular identity of the exosite. Most likely the exosite is not too far from the active site and may be composed of both NCT, TM2, and loop 1 of PS. The exosite may be mapped by blocking substrate entry into the GS active site using an active site GSI. Disulfide gates may be engineered to probe the exosite and substrate translocation pathway, as was carried out with rhomboids (Baker et al., 2007).
2. From the exosite, C99 translocates to the enzyme active site, forming an intermolecular β-sheet with PS (Zhou et al., 2019). We do not know the pathway of substrate translocation, partly because we do not know the exact substrate docking site. TM2 and TM6 are the most dynamic TMs in PS1 and therefore are mostly likely involved in the lateral gating mechanism of substrate translocation. The detailed dynamics of substrate translocation can be elucidated by combining the power of molecular dynamics simulations and cutting-edge experimental structural determination methods for membrane proteins.
3. Initial ε-cleavage occurs at T48 or L49, releasing the AICD and forming Aβ48 or Aβ49, precursor peptides of the Aβ42 or Aβ42 production line, respectively. Here, the catalytic mechanism is not known, nor how the two active site aspartates coordinate a catalytic water molecule to facilitate hydrolysis. In all of the solved structures of GS and MCMJR1, the catalytic aspartates appear to be too far away from each other to coordinate a catalytic water. Thus, we have yet to capture the conformation of the GS active site in a catalytically competent state. Because of the stability of hybrid β-sheet at the C-terminus of C83, a large conformational change is needed for reducing this intermolecular interaction to facilitate the release of AICD. How this happens also remains an open question.
4. Following ε-cleavage, carboxypeptidase activity of GS trims Aβ48/Aβ49 processively (Figure 10), shedding tripeptides to produce Aβ42 and Aβ40. The mechanism of processive cleavage is not known. Based on biochemical evidence, Wolfe et al. proposed a tripeptide binding pocket in the GS active site for P1′P2′P3′ (Wolfe, 2020), which is not obvious in the GS–C83 complex. How the active site aspartates get to the next cleavage site on the substrate, as well as the driving force for this process, is not clear. It is straightforward to speculate that it involves concerted conformational changes and dynamics in both GS and the substrate. The catalytic aspartates in PS may move towards more N-terminal cleavage sites in APPTM while GS continues to unwind the substrate. The timing of AICD release and C-terminal trimming is not clear, for example, whether they are concurrent, sequential, or of random order. We suggest that MD simulations will be extremely helpful in providing clues for experimentalists in this area.
5. Finally, following processive cleavage by GS, the shorter and more hydrophilic Aβ fragment dissociates from the enzyme and exits the membrane. The mechanism of Aβ peptide or AICD release has been little studied. What are the kinetics and pathway of Aβ release? How does it involve NCT and other components of GS? During processive cleavage, Aβ fragments may either be released or undergo one more step of trimming (e.g., Aβ42 is released vs. Aβ42 is cut down to Aβ38). How is this bifurcation in the Aβ production pathway determined mechanistically? Both equilibrium (Szaruga et al., 2017) and kinetic stability of the Aβ/GS complex might be critical determinants in this situation. Answers to these questions have important implications for the design and discovery of new GSMs and selective GSIs.

Given the recent structural insights, an intriguing question for AD drug discovery is whether selective GSIs can be designed or discovered. Yang et al. (2019) pointed out several distinct pockets in the GS–C83 complex that have different shapes and dimensions compared with the GS–Notch complex (Zhou et al., 2019), which may be targeted for rational drug design. However, it is important to note that GS is highly dynamic, and binding pockets can stretch and/or shrink. Thus, for selective GSI, we may still need to rely on docking coupled with long-time-course MD simulation, high-throughput (HT), or ultra-HT methods such as DNA encoded libraries which enable screening of tens of billions of compounds in a single test tube (Satz, 2018).

## Conclusion

There has been tremendous progress in the structural and mechanistic investigation of the substrate–enzyme interaction in intramembrane proteolysis, especially in light of the recent cryo-EM structures of GS–C83 and GS–Notch complexes. In particular, cryo-EM revealed the formation of a hybrid, intermolecular β-sheet between GS and its substrates, which is consistent with numerous biochemical and biophysical studies. However, our knowledge of how GS interacts with its substrates, which is crucial for developing selective amyloid reduction agents, remains far from complete.

## Author Contributions

XL and CW prepared the text and figures. JZ, YZ, IU-B, SF, and RL edited and revised the manuscript.

## Conflict of Interest

The authors declare that the research was conducted in the absence of any commercial or financial relationships that could be construed as a potential conflict of interest.

## Abbreviations

I-CLiPs: intramembrane-cleaving proteases
GS: γ-secretase
APP: amyloid precursor protein
cryo-EM: cryo-electron microscopy
SREBPs: sterol regulatory element-binding proteins
AD: Alzheimer’s disease
S2P: site-2-protease
S1P: site-1-protease
TM: transmembrane helix
IAP: intramembrane aspartate protease
PS: presenilin
SPP: signal peptide peptidase
TMD: transmembrane domain
PSH: presenilin homolog
NTF: amino-terminal fragment
CTF: carboxy-terminal fragment
SANS: small angle neutron scattering
Rce1: Ras and a-factor converting enzyme 1
MmRce1: *Methanococcus maripaludis* homolog of Rce1
ZMPSTE24: zinc metallopeptidase STE24
Aβ: amyloid-β peptide
APPTM: transmembrane domain of APP
C99: C-terminal fragment containing 99 amino acid residues
AICD: APP intracellular domain
FAD: familial Alzheimer’s disease
PSEN1 and PSEN2: presenilin 1 and 2 genes
NICD: Notch intracellular domain
NCT: nicastrin
APH-1: anterior pharynx-defective 1
PEN-2: presenilin enhancer 2
DpNCT: *Dictyostelium purpureum* homolog of nicastrin
HsNCT: human nicastrin
ECD: extracellular domain
CSP: chemical shift perturbation
GSIs: γ-secretase inhibitors
dUVRR: deep-ultraviolet resonance Raman spectra.
